# Effects of E2 on the IDO1‐mediated metabolic KYN pathway in OVX female mice

**DOI:** 10.1111/jcmm.70179

**Published:** 2024-10-28

**Authors:** Xi Jiang, Xuefeng Yu, Shuran Hu, Huidan Dai, Hanqin Zhang, Yuyang Hang, Xupei Xie, Yubo Yang, Fan Wu

**Affiliations:** ^1^ Department of Pharmacy Zhejiang University Mingzhou Hospital Ningbo China; ^2^ Department of Pharmacy Zhejiang Pharmaceutical University Ningbo China; ^3^ Department of Neurosurgery South Yunnan Central Hospital of Yunnan Province (The First People's Hospital of Honghe Prefecture) Mengzi China; ^4^ Department of Pharmacy Shaoxing Second Hospital Shaoxing China; ^5^ Department of Pharmacy The First Affiliated Hospital of Zhejiang Chinese Medical University (Zhejiang Provincial Hospital of Chinese Medicine) Hangzhou China

**Keywords:** 17β‐estradiol (E2), ERα, ERβ, IDO1, neuroinflammation, oxidative stress

## Abstract

The aim of this study was to investigate the role of 17β‐estradiol (E2)‐mediated oestrogen receptor (ER) in modulating the depressive‐like behaviours of ovariectomy (OVX) mice and the associated mechanisms. E2 was administrated in OVX mice. The behaviour and physiological changes of OVX mice including immobility time in tail suspension test (TST) and forced swimming test (FST), levels of serum E2, inflammatory mediators, oxidative stress factors, indoleamine2,3‐dioxygenase 1 (IDO1) and the neurotransmitters mediated by IDO1 activation were then recorded. Cell injury models established by lipopolysaccharide (LPS) or H_2_O_2_ stimulation in HT22 and BV2 cells were employed to further explore the mechanisms of E2's function. E2 treatment improved OVX‐induced increase of immobility time in FST and TST. Meanwhile, E2 ameliorated the changes of inflammatory factors (NF‐κB, TNF‐α and IL‐6), IDO1, IDO1‐mediated TRP/KYN pathway and oxidative stress factors (iNOS, MDA, GSH and SOD) in the hippocampus of OVX mice. Interestingly, ERβ inhibitor abolished E2's inhibitory effects on the inflammation and IDO1‐mediated TRP/KYN pathway; ERβ inhibitor also abolished E2's anti‐oxidative stress effect. In cell experiments, ERβ small interfering RNA (siRNA) pretreatment reversed E2's anti‐inflammatory effect on LPS‐treated HT22 and BV2 cells and E2's inhibitory effect on IDO1 expression in LPS‐treated BV2 cells. ERβ siRNA pretreatment also reversed E2's anti‐oxidation effect on H_2_O_2_‐treated HT22 cells. E2 exert the antidepressant function in OVX mice via ERβ‐modulated suppression of NF‐κB‐mediated inflammatory pathway, oxidative stress factors and IDO1‐mediated TRP/KYN pathway in the hippocampus.

## 1 INTRODUCTION

Female individuals are more sensitive to stress due to fluctuations in oestrogen levels. In elderly individuals, the level of oestrogen is low, causing elderly women to lose the protective effects of oestrogen; therefore, postmenopausal elderly women are especially prone to depression.^1^, ^2^ Clinical and animal studies have shown that oestrogen replacement therapy can improve depressive symptoms caused by reduced hormone levels. However, oestrogen has extensive physiological effects because of the widely distributed oestrogen receptors (ER), for example, Erα and Erβ. ERα is mainly distributed in the hypothalamus and pituitary, and mainly affects pituitary hormones and metabolic functions; ERβ is distributed in the cortex, hippocampus and amygdala, and is mainly involved in functions such as central emotion, memory and cognition.^3^, ^4^ Long‐term administration of oestrogen increases the risk of obesity, addiction and cancer.^5^, ^6^ Thus, a better understanding of the ER and the relevant mechanisms by which ER exerts antidepression effects is conducive to identifying effective and safe therapeutic targets.

Central nervous system inflammation is one of the main pathological mechanisms of depression.^7^ Clinical autopsy results have shown that there is a plenty of activated microglia in the hippocampus and prefrontal cortex of suicide patients with depression^8^; these activated microglia lead to significant increases in the levels of inflammatory mediators in these brain regions, indicating that the brains of the patients with depression are in an inflammatory state. Animal studies have shown that in rat models of depression induced by chronic stress, there are also a lot of activated microglia in the hippocampal region, resulting in an abundance of inflammatory mediators, such as tumour necrosis factor alpha (TNF‐α) and interleukin‐6 (IL‐6).^9^ Interestingly, oestrogen is associated with neuroinflammation, and oestrogen withdrawal leads to microglia activation, which in turn increases the expression of inflammatory mediators.^10^ When the central nervous system is in the inflammatory state, the overexpressed neuroinflammatory mediators leads to a series of pathological changes, such as the activation of indoleamine 2,3‐dioxygenase (IDO).^11^ IDO is a key enzyme in the metabolism of L‐tryptophan (TRP) in the kynurenine (KYN) pathway and regulates the associated neurotoxic substances such as quinolinic acid (QA). IDO is a critical role of the inflammatory factor hypothesis of depression, the monoamine hypothesis of depression and neurotoxicity hypothesis of depression.^11^ There are three subtypes of IDO, namely, IDO1, IDO2 and TDO. Among them, IDO1 is widely distributed in the brain and has the strongest effect on the KYN pathway.^12^ Previous studies found that E2 can improve inflammation‐induced depression‐like behaviour by suppressing neuroinflammation.^13^ However, the relationship between E2 and the IDO‐mediated KYN metabolic pathway remains unclear.

Oxidative stress injury is also one of the main pathological mechanisms of depression.^14^ In rat models of depression, the levels of the oxidative stress indicators including inducible nitric oxide synthase (iNOS) and malondialdehyde (MDA) in the hippocampal region significantly increased, and the levels of superoxide dismutase (SOD) and glutathione (GSH) decreased,^15^ indicating that depressive behaviour is significantly related to oxidative stress damage in the hippocampal region. Oestrogen also plays a role in the regulation of oxidative stress. Studies have shown that oestrogen withdrawal can increase the level of MDA and decrease the levels of SOD and GSH in the hippocampus,^16^ and administration of exogenous oestrogen (17β‐estradiol) can significantly improve the oxidative stress induced by oestrogen withdrawal.^17^ Whereas the specific regulatory mode of oestrogen‐ER in oxidative stress remains unclear.

The present study established an oestrogen withdrawal‐induced depressive animal model by conducting ovariectomy (OVX) on mice to investigate: (1) whether the occurrence of oestrogen withdrawal‐induced depression is related to the level of IDO1 in the hippocampus; (2) the effects of E2 on the OVX‐induced changes in mice and the role of ER thereof; (3) whether the possible function of E2‐ER related to the modulation of depression is difference between females and males.

## 2 MATERIALS AND METHODS

### 2.1 Animals and cells

In the animal experiments, female and male ICR mice (20‐25 g, 10 weeks old) were purchased from the Experimental Animal Center of Wenzhou Medical University. The mice were housed in a room with standard laboratory conditions (temperature: 25 ± 1°C; humidity: 40%–60%). All the animal experiments were approved by the Committee on Animal Care and Use of Zhejiang Pharmaceutical University (Approval number: zyll202303001) and conducted in accordance with the National Institutes of Health Guide for Care and Use of Laboratory Animals.

In the cell experiments, BV2 and HT‐22 cells were purchased from Cell Storage Center, Wuhan, China. The cells were cultured using Dulbecco's modified Eagle's serum (DMEM; Life Technologies, Carlsbad, CA) supplemented with 10% foetal bovine serum (FBS; Life Technologies), GlutaMAX (Life Technologies) and antibiotic–antimycotic (Life Technologies).

### 2.2 Experimental design

The experiments in this study consisted of four parts.

#### 2.2.1 Part 1

This part is to establish an ovariectomy (OVX) mice model and explore the subsequently changes in mice. Twenty‐four female mice were randomly divided into 3 groups: sham group, 3‐day post‐OVX group and 7‐day post‐OVX group (*n* = 8 per group). The mice in the sham group received the same surgery except for OVX. The mice in each group were sacrificed after the behaviour tests, and the blood and hippocampal tissue samples were collected.

#### 2.2.2 Part 2

This part is to investigate the function of 17β‐estradiol (E2) on the OVX mice. E2 (sigma), IDO1 inhibitor 1‐MT (sigma), oestrogen receptor α (ERα)‐selective antagonist MPP (sigma) and ERβ‐selective antagonist R, RTHC (Tocris) were used. Seventy‐two female mice were randomly divided into 6 groups: sham group, OVX group, OVX + E2 (0.5 mg/kg, vehicle: 2% DMSO, i.p.) group, OVX + 1‐MT (9 mg/kg, vehicle: sesame oil, i.p.) group, OVX + E2 + MPP (2.0 mg/kg, vehicle: 2% DMSO, i.p.) group and OVX + E2 + R, RTHC (0.1 mg/kg, vehicle: 2% DMSO, i.p.) group. The doses of E2, 1‐MT, MPP and R, R‐THC were selected based on our previous studies.^3^, ^18^ Each group contains 12 mice, 6 for the forced swim test (FST) and another 6 for TST. The drug administration was performed each day and lasted for 1 week before the behavioural tests. After the behavioural tests, the mice were sacrificed, and the hippocampal tissues were collected.

#### 2.2.3 Part 3

This part is to investigate the function of E2 in nerve cells to elucidate the underlying mechanism of E2's effects on OVX mice. Microglial cells BV2 and hippocampal neuro cells HT‐22 were employed and transfections of ERα and ERβ siRNA (Santa Cruz Biotechnology) were performed. The BV2 cells were cultured with E2 (100 nM) and ERα or β small interfering RNA (siRNA) following a 24‐h LPS (μg/mL) treatment to explore the effect of E2‐ERα/β on LPS‐induced neuroinflammation in BV2 cells. The HT‐22 cells were cultured with E2 (100 nM) and ERα or β siRNA for 24‐h following a 24‐h LPS (5 μg/mL) or H_2_O_2_ (15 μg/mL) treatment to explore the effects of E2‐ERα/β on LPS‐induced neuroinflammation and H_2_O_2_‐induced oxidative stress in HT‐22 cells. The dose of E2 (100 nM) was chosen based on our previous study.^3^ The dose of LPS (1 μg/mL) used in BV2 cells was chosen based on a previous study^19^; The doses of LPS and H_2_O_2_ used in HT‐22 cells were chosen based on the tolerance tests.

For the siRNA transfections, lipofectamine 2000 (Invitrogen, USA) was used as the transfection reagent according to the transfection requirements. HT‐22 cells or BV2 cells were incubated with a concentration of 20 nM siRNA in the presence of transfection reagent. After transfection, cells were collected for subsequent experiments.

For the assessment of cell survival rate, a MTT kit (Abcam, Shanghai, China) was used. In brief, cells were incubated in the culture medium with 20 μL MTT solution for 4 h. Then, 100 μL DMSO was added in per hole. After incubation in cell incubator for 15 min, the absorbance of each well was measured at 490 nm wavelength by a spectrophotometer (Thermo Scientific, USA).

#### 2.2.4 Part 4

This part is to investigate whether the function of E2 is different between males and females. Forty male mice were divided into five groups: control group, LPS (1.8 mg/kg, vehicle: saline, i.p.) group and LPS+ E2 (0.5, 5 and10 mg/kg, i.p.) groups (*n* = 8 per group). The concentration of LPS used in mice was determined based on our previous study.^20^ The mice in the control group were injected with an equal volume of saline. The experiment lasted for 1 week. E2 was given for 7 consecutive days. The injection of LPS was performed at the 6th day, and the behaviour performance of the mice were assessed 24 h post‐injection of saline or LPS.

### 2.3 Ovariectomy (OVX) mice model

The mice were anaesthetised by intraperitoneal injection of 30 mg/kg sodium pentobarbital solution. Afterwards, the mice were placed in the prone position, and then shaving was performed on both sides of the waist. Then, the peritoneum was cut open to explore the abdominal cavity, and the ovaries located along the uterus forward at both sides were removed. Penicillin (2.5 × 105 U/kg, intramuscular injection, sigma) was administered postoperatively to prevent infection. The mice in the sham group underwent the same surgical operations except for the ovarian removal.

### 2.4 Forced swim test (FST)

The mice were placed in a round swimming tank of 20 cm in diameter and 45 cm in height. The water inside the tank was 20 cm in depth and the temperature was maintained at (22 ± 1)°C. The mice acclimatized the environment inside the tank for 2 min, and the accumulated immobility time of the mice during the subsequent 4 min was recorded.

### 2.5 Tail suspension test (TST)

The mice were suspended 50 cm over the ground by fixing the tail using adhesive tape at 1 cm away from the tip of the tail. The mice were acclimatized to the situation for 2 min, and the accumulated immobility time of the mice during the subsequent 4 min was recorded.

### 2.6 Locomotor activity

Spontaneous locomotor activity (SLA) is the observed behaviour of an animal in its familiar environment. Since various physical and/or psychological changes can affect the SLA, it is a widely used index to assess animal conditions in various research fields.^21^ In our study, the assessment of locomotor activity was carried out as previously described.^22^ Briefly, locomotor activity of mouse was measured by an experimental instrument with five activity chambers (JZZ98, Institute of Materia Medica, Chinese Academy of Medical Sciences, China). Mice were placed in the chambers and their paws contacted or disconnected the active bars producing random configurations that were converted into pulses. The pulses, which were proportional to the locomotor activity of the mice, were automatically recorded as the cumulative total counts of motor activity. Mice performed a training session for 5 min (pre‐test), and then locomotion counts were recorded during the 10‐min testing period. Each mouse received locomotor a activity test before FST or TST.

### 2.7 High Performance Liquid Chromatography (HPLC)^18^

The concentrations of KYN, TRP, 5‐hydroxytryptamine (5‐HT), 5‐hydroxyindolacetic acid (5‐HIAA), 3‐hydroxykynurenine(3‐HK), kynurenic acid (KA) and quinolinic acid (QA) in hippocampal tissues were analysed by HPLC. Each hippocampal tissue sample was weighed and homogenized by ultrasonication in a mixed solution of 0.1 N HClO_4_ and 25 μM ascorbate. The homogenate was centrifuged at 12000 r/min for 20 min at 4°C.

For KYN and TRP, the supernatant was extracted and loaded into a Costar Spin‐X centrifuge tube filter (0.22 μm), and then 20 μL filtered supernatant was taken for HPLC analysis. The mobile phase (pH = 4.6) consists of 75 mM NaH_2_PO_4_, 25 μM EDTA (disodium salt) and 100 μL/L triethylamine diluted in acetonitrile/water (v/v 6:94) solution.

For 5‐HT, 5‐HIAA, 3‐HK, KA and QA, the supernatant and a solution containing 0.2 M potassium citrate, 0.3 M dipotassium hydrogen phosphate and 0.2 M EDTA were mixed in a ratio of 2:1, and then the mixed liquid was centrifuged at 12,000 rpm for 20 min at 4°C. The supernatant was extracted and loaded into a Costar Spin‐X centrifuge tube filter (0.22 μm), and then 20 μL filtered supernatant was taken for HPLC analysis. The mobile phase (pH = 3.0) consists of 75 mM NaH_2_PO_4_, 25 μM EDTA, 0.45 mM octane sulfonic acid and 100 μL/L triethylamine diluted in acetonitrile/water (v/v 6:94) solution.

### 2.8 Enzyme‐Linked Immunosorbent Assay (ELISA)^23^

The levels of E2 in the blood and the levels of iNOS, nuclear factor kappa‐B p65 (NF‐κBp65), IL‐6 and TNF‐α in the hippocampal tissue and BV2 cells were detected by an ELISA kit (Thermo Scientific, USA). The expression of IDO1 in the blood and hippocampus were estimated by an ELISA kit (Cusabio, China). The levels of ERα and ERβ in the hippocampus were estimated by an ELISA kit (Spbio, China). Briefly, diluted protein standards and samples were added to a 96‐well ELISA plate, followed by biotinylated antibodies. After washing with wash buffer, the prepared solution of avidin and horseradish peroxidase‐conjugated complex was added to each well. Finally, the reaction was terminated by the stopping solution. For E2, IL‐6, IDO1, TNF‐α, iNOS, ERα and ERβ the optical density (OD) values were measured at 450 nm. For NF‐κBp65, the OD value was assessed at 405 nm.

### 2.9 Measurement of Oxidative‐Stress Markers

The concentrations of GSH, MDA and SOD in the hippocampal tissue and HT‐22 cell samples were measured by the corresponding reagent kits (Thermo Scientific). The protocol for the measurements was based our previous studies.^23^, ^24^ The MDA and GSH content were expressed as nmol/mg protein. The SOD content was expressed as U/mg.

### 2.10 Statistical Analysis

The data were expressed as mean ± SD. Statistical analysis was performed by one‐way ANOVA, and the Tukey test was used for multiple comparisons of data among groups. *p* < 0.05 was considered as a significant difference.

## 3 RESULTS

### 3.1 Correlation analysis between depressive‐like behaviour and levels of serum E2 and hippocampal IDO1 in OVX mice

**FIGURE 1:**
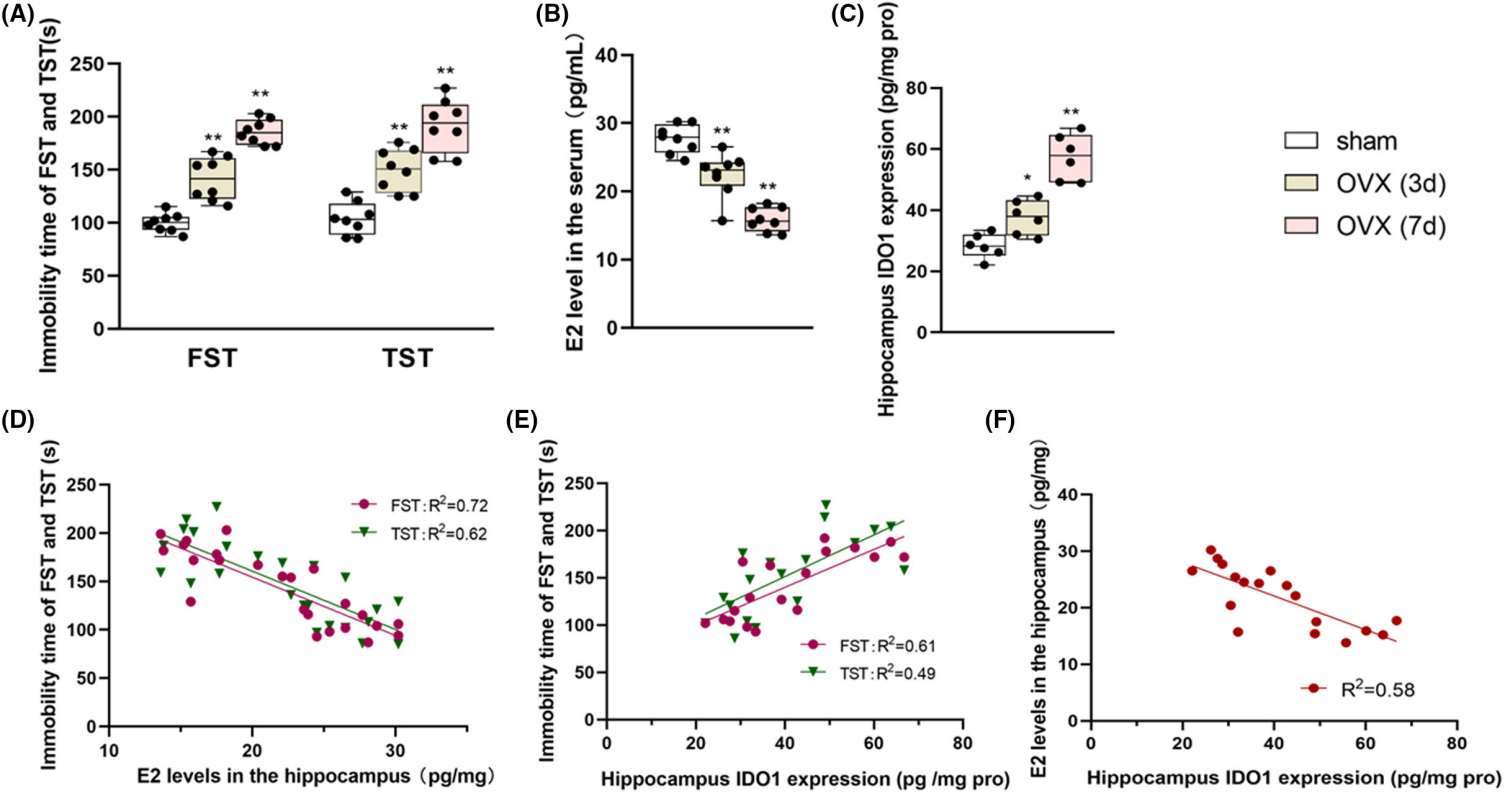
The immobility time in FST and TST (A), E2 level in the serum (B) and IDO1 level (C) in the hippocampus. Correlation analysis between immobility time in depressive behaviours and E2 orIDO1 levels (D, E). Correlation analysis between E2 level and the IDO1 level in the hippocampus (F). Values are expressed as the mean ± S.D. with 8 mice in each group. *p < 0.05 and **p < 0.01 versus the sham group.

As shown in Figure 1A, both in the FST and TST, the immobility time of the mice in the 7‐day post‐OVX group were significantly longer than that in the sham group Meanwhile, the serum E2 level was significantly deceased (*p* < 0.001, Figure 1B) and the IDO1 level in the hippocampus was significantly increased (*p* < 0.001, Figure 1C) in the mice of the 7‐day post‐OVX group. The correlation analysis indicated that OVX‐induced depressive‐like behaviour was negatively correlated with serum E2 level (*R* ^2^ = 0.72 for FST, *R* ^2^ = 0.62 for TST, Figure 1D) and positively correlated with hippocampal IDO1 level (*R* ^2^ = 0.61 for FST, *R* ^2^ = 0.49 for TST, Figure 1E). Serum E2 level was negatively correlated with hippocampal IDO1 level (*R* ^2^ = 0.58, Figure 1F).

### 3.2 Effects of E2 on OVX‐induced depression‐like behaviour in female mice

**FIGURE 2:**
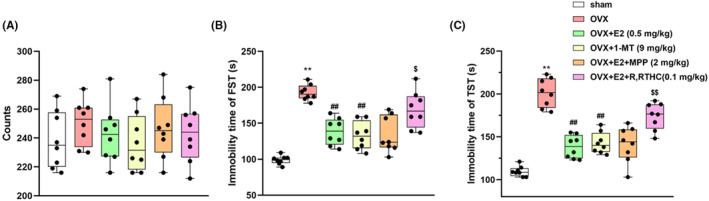
Effects of E2 on locomotor activity (A), forced swimming test (B) and tail suspension test (C) in OVX mice. Values are expressed as the mean ± S.D. with 8 mice in each group. **p < 0.01 and compared with the sham group; ##*p* < 0.01 compared with the OVX group. $*p* < 0.05 and $$*p* < 0.01 compared with the E2‐treated OVX group.

As shown in Figure 2B,C, E2 administration significantly ameliorated OVX‐induced increase of the immobility time in FST and TST (*p* < 0.01 for both FST and TST). Interestingly, the administration of the IDO1 inhibitor 1‐MT also present a similar effect as the exogenous E2. The antidepressant effect of E2 was reversed by the ERβ antagonist R‐RTHC (*p* < 0.05 for FST and *p* < 0.01 for TST), but not the ERα antagonist MPP. The data in Figure 2A demonstrated that there was no significant difference in the spontaneous activity of animals among all the treatment groups.

### 3.3 Effects of E2 on hippocampal inflammatory factors and IDO1 in OVX female mice

As shown in Table 1, the levels of the inflammatory factors significantly increased in the hippocampal tissue of OVX mice (*p* < 0.01 for NF‐κB p65, TNF‐α and IL‐6). E2 treatment alleviated OVX‐induced overexpression of inflammatory factors (*p* < 0.01 for NF‐κB p65, TNF‐α and IL‐6); co‐treatment with the ERβ antagonist R‐RTHC reversed the anti‐inflammatory effect of E2 (*p* < 0.05 for NF‐κB p65 and IL‐6, *p* < 0.01 for TNF‐α), while the ERα antagonist MPP had no significant effect.

**TABLE 1:** Effects of E2 on NF‐κBp65, IL‐6 and TNF‐α expressions in OVX mice.

Group	NF‐κBp65 (ng/mg)	IL‐6 (pg/mg)	TNF‐α (pg/mg)
Sham	15.4 ± 1.8	24.6 ± 2.3	7.5 ± 1.2
OVX	28.3 ± 3.4^a^	46.1 ± 4.2^a^	14.7 ± 2.3^a^
OVX + E2	20.4 ± 3.4^b^	34.4 ± 3.5^b^	10.4 ± 1.5^b^
OVX + 1‐MT	24.9 ± 4.1	43.1 ± 6.2	14.1 ± 0.8
OVX + E2 + MPP	21.1 ± 2.6	37.4 ± 4.3	9.5 ± 1.4
OVX + E2 + R, RTHC	27.4 ± 2.5^c^	44.0 ± 4.7^c^	14.2 ± 1.8^d^

*Note*: Values are expressed as mean ± S.D. *n* = 6. Data analysis was performed using Tukey's Test.

^a^ *p* < 0.01 versus the sham group.

^b^ *p* < 0.01 versus the OVX group.

^c^ *p* < 0.05 versus the OVX + E2 group.

^d^ *p* < 0.01 versus the OVX + E2 group.

**FIGURE 3:**
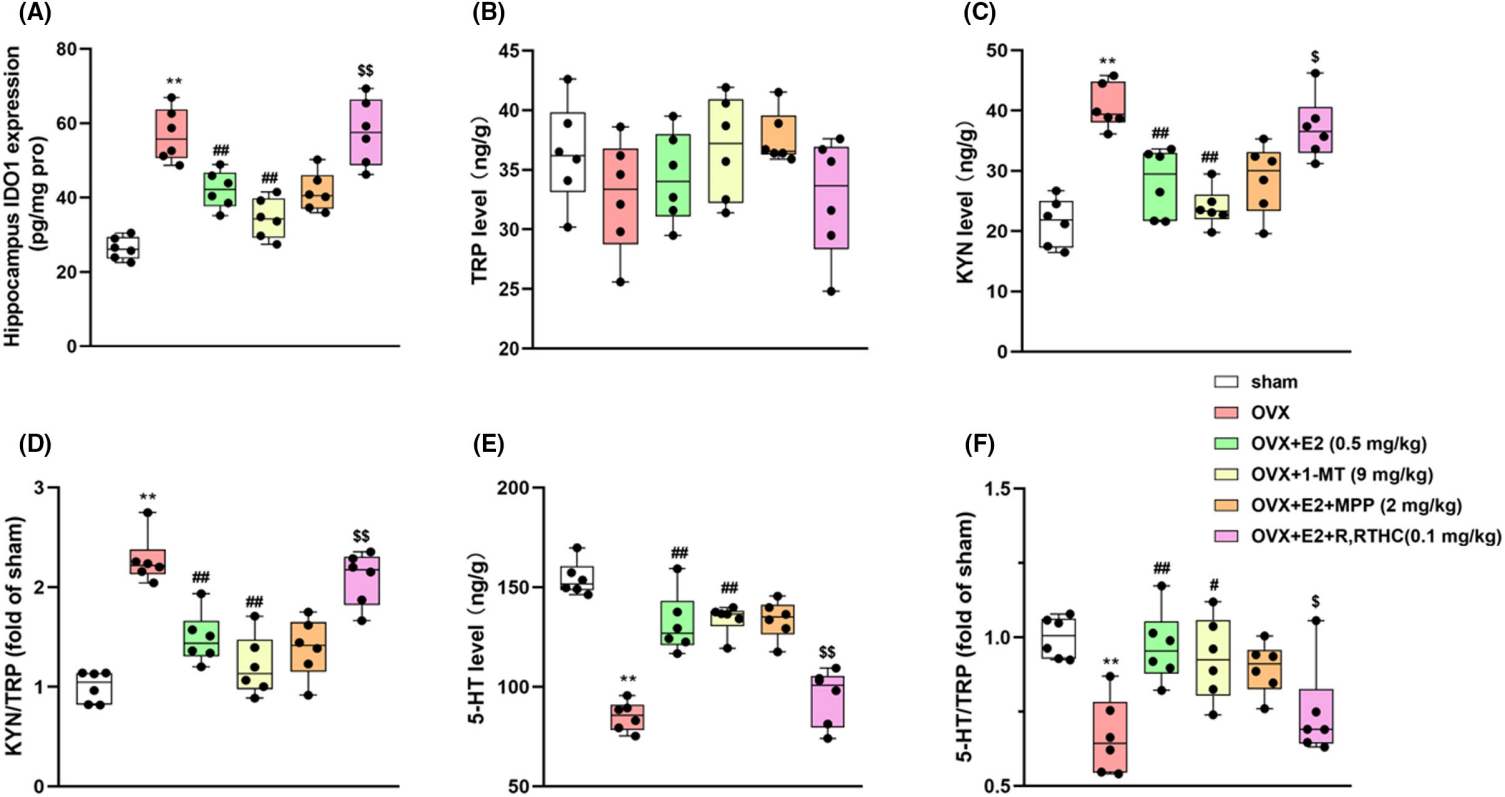
Effects of E2 on the IDO1 level (A), L‐tryptophan (TRP) level (B), kynurenine (KYN) level (C), KYN/tryptophan (TRY) ratio (D), serotonin (5‐HT) level (E), 5‐HT/TRP ratio (F) in the hippocampus of th OVX mice. Values are expressed as the mean ± S.D. with 6 mice in each group. **p < 0.01 compared with the sham group; ^#^ *p* < 0.05 and ^##^ *p* < 0.01 compared with the OVX group. ^$^ *p* < 0.05 and ^$$^ *p* < 0.01 compared with the E2‐treated OVX group.

As for the expression of hippocampal IDO1, Figure 3 showed that E2 and 1‐MT significantly reduced the OVX‐induced increase of IDO1 activity (*p* < 0.01 for both E2 and 1‐MT, Figure 3A), and co‐treatment of ERβ antagonist R‐RTHC reversed the inhibitory effect of E2 on IDO1 expression (*p* < 0.01).

### 3.4 Effects of E2 on the IDO1‐mediated metabolic KYN pathway in OVX female mice

**FIGURE 4:**
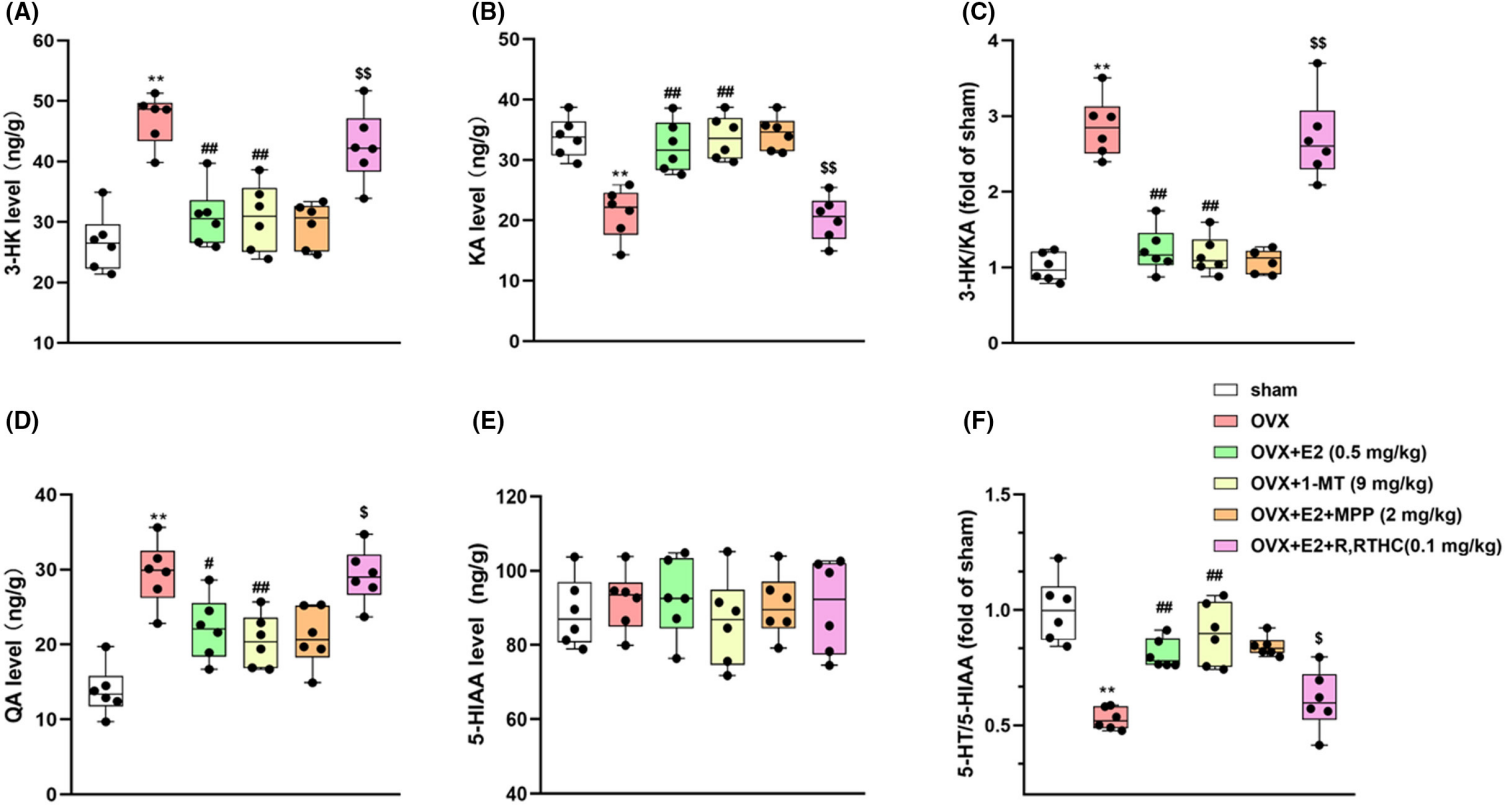
Effects of E2 on the 3‐hydroxykynurenine (3‐HK) level (A), kynurenic acid (KA) level (B), 3‐HK/KA ratio (C), quinolinic acid (QA) level (D), 5‐hydroxyindoleacetic acid (5‐HIAA) levels (E) and 5‐HIAA/5‐HT ratio (F) in the hippocampus of the OVX mice. Values are expressed as the mean ± S.D. with 6 mice in each group. **p < 0.01 compared with the sham group; ^##^ *p* < 0.01 compared with the OVX group. ^$^ *p* < 0.05 and ^$$^ *p* < 0.01 compared with the E2‐treated OVX group.

In mice received OVX surgery, the hippocampal KYN level as well as the KYN to TRP ratio significantly increased (ps <0.01, Figure 3C,D); the 5‐HT level and the 5‐HT to TRP ratio significantly decreased (*p* < 0.01 s, Figure 3E,F); the levels of KA decreased (*p* < 0.01, Figure 4B), 3‐HK and 3‐HK to KA ratio significantly increased (*p*s < 0.01, Figure 4A,C). QA, a downstream metabolite of 3‐HK, also increased (*p* < 0.01, Figure 4D). For the levels of 5‐HT and 5‐HIAA, the ratio of 5‐HT to 5‐HIAA significantly decreased (*p* < 0.01, Figure 4F), while no change was found in the level of 5‐HIAA (Figure 4E) in the OVX mice. This indicated that E2 increased the ratio of 5‐HT to 5‐HIAA by increasing the level of 5‐HT. The changes in KYN level, KYN to TRP ratio, 5‐HT level, 5‐HT to TRP ratio, 3‐HK, KA, 3‐HK to KA ratio and QA levels were relieved by E2 treatment. However, ERβ antagonist R, RTHC reversed the effect of E2 on IDO1‐mediated KYN pathway metabolism (Figure 3C–F and Figure 4A–F). The ERα antagonist MPP had no such effect.

### 3.5 Effects of E2 on the expression of oxidative stress factors in OVX female mice

**FIGURE 5:**
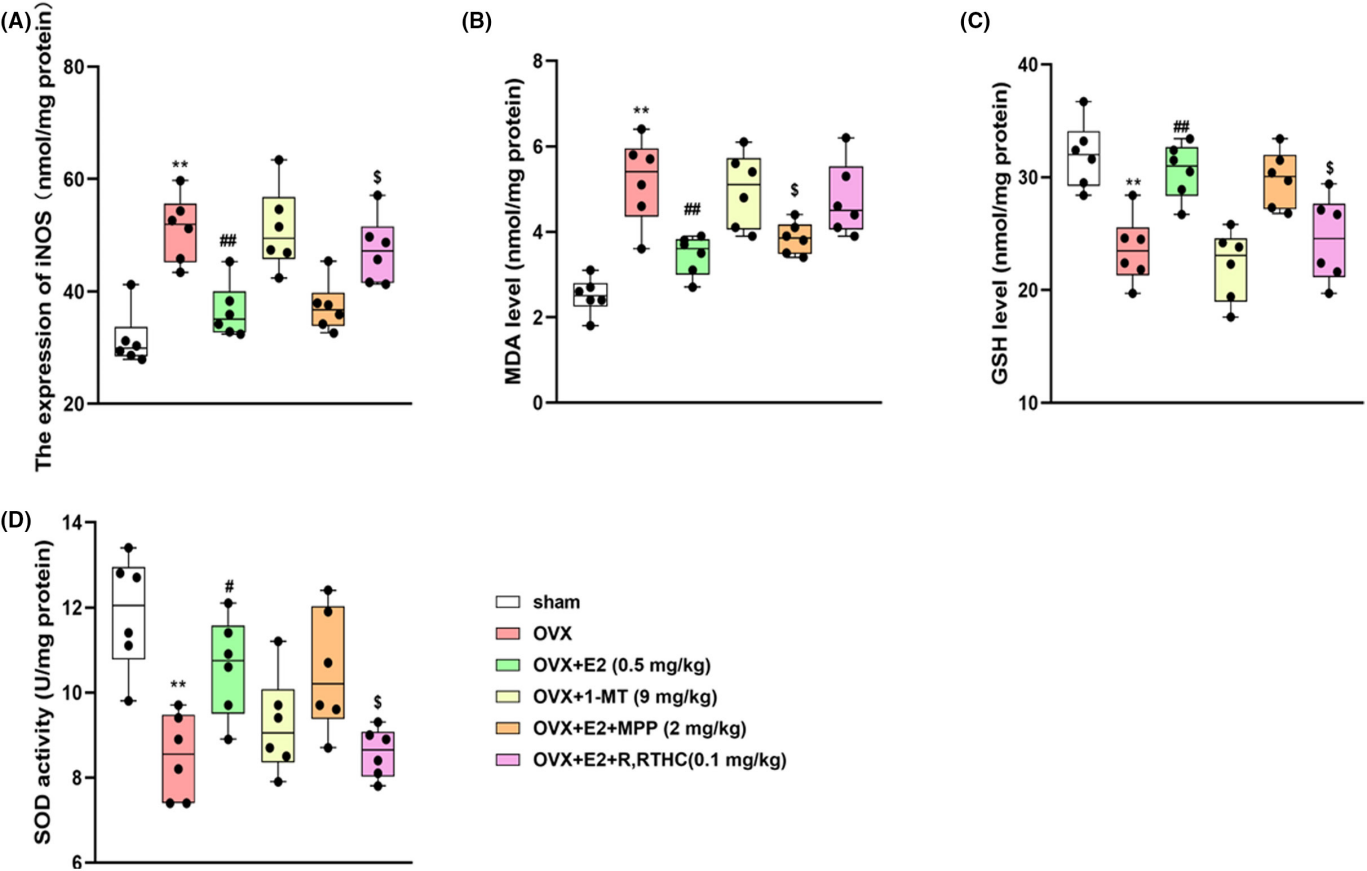
Effects of E2 on the expression of iNOS (A), MDA (B), GSH (C) and SOD (D) in the hippocampus of the OVX mice. Values are expressed as the mean ± S.D. with 6 mice in each group. **p < 0.01 compared with the sham group; ^#^ *p* < 0.05 and ^##^ *p* < 0.01 compared with the OVX group. ^$^ *p* < 0.05 compared with the E2‐treated OVX group.

As shown in Figure 5, OVX surgery induced increases in iNOS (*p* < 0.01, Figure 5A) and MDA levels (*p* < 0.01, Figure 5B) and decreases in GSH (*p* < 0.01, Figure 5C) and SOD levels (*p* < 0.01, Figure 5D) in the hippocampus. E2 significantly ameliorated OVX‐induced oxidative stress by normalizing iNOS (*p* < 0.01), MDA (*p* < 0.01), GSH (*p* < 0.01), and SOD levels (*p* < 0.05). The antioxidative effect of E2 was reversed by the ERβ antagonist, but not the ERα antagonist.

### 3.6 Effects of ERα/β siRNA on the LPS‐induced inflammatory response and IDO1 expression in BV2 cells

**FIGURE 6:**
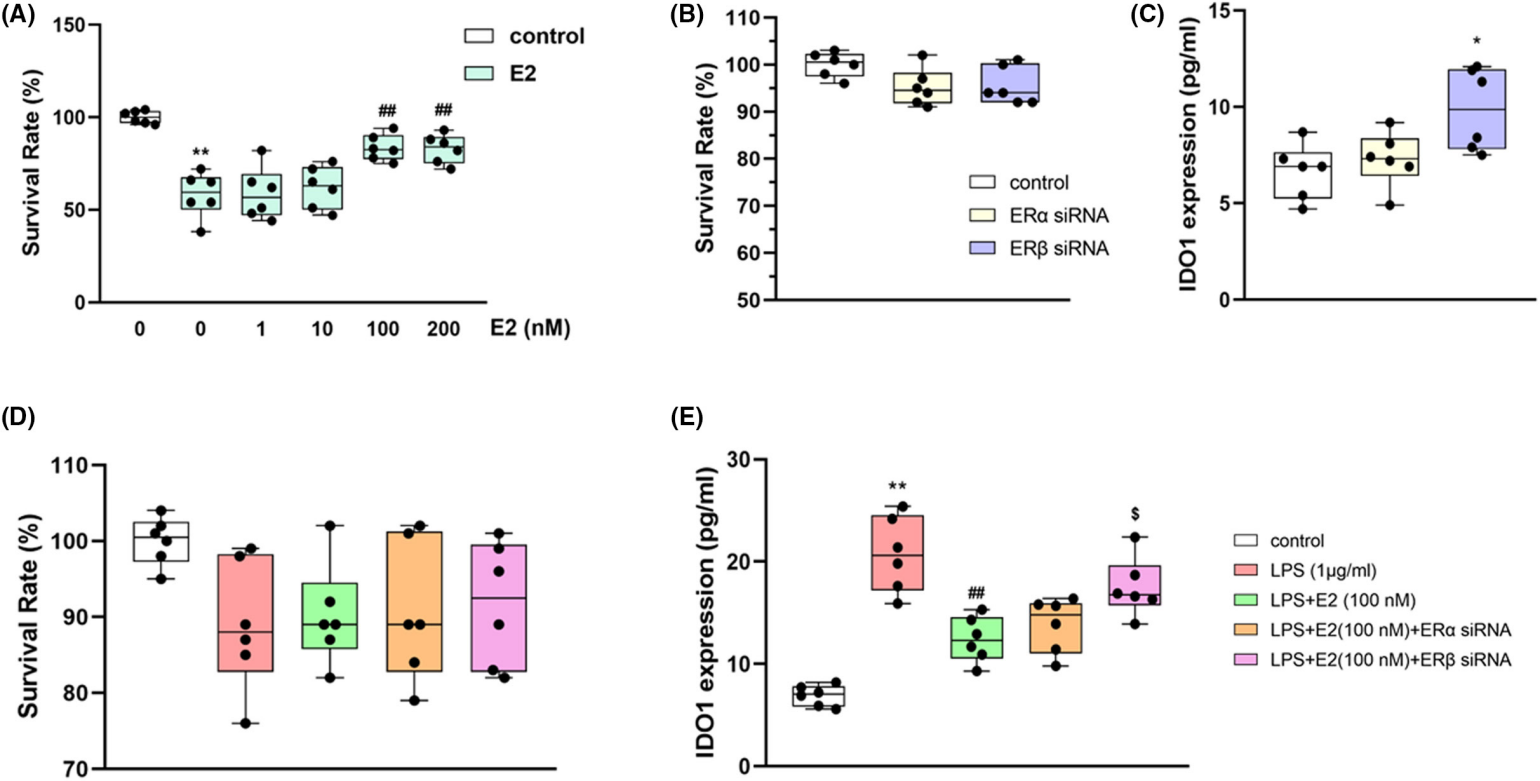
E2 treatment (10, 50, 100, 150, 200 mM) did not affect the survival rate of BV2 cells (A). ERα/β siRNA treatment did not affect the survival rate of BV2 cells (B). No significant differences of the survival rate were observed between the control, LPS, LPS + E2, LPS + E2+ ERα/β siRNA groups (C). E2 treatment inhibited the LPS‐induced increase in IDO1 levels of BV2 cells (D) while ERβ siRNA reversed E2's function. ERβ siRNA alone increased the IDO1 level in BV2 cells (E). Values are expressed as the mean ± S.D. with 6 mice in each group. **p < 0.01 compared with the control group; ^##^ *p* < 0.01 compared with the LPS group. ^$^ *p* < 0.05 compared with the E2‐treated LPS group.

Figure 6A–6C demonstrated that the concentrations of E2 (100 nM), siRNA (20 nM), and LPS (1 μg/mL) used in this study is non‐toxic to BV2 cells. When BV2 cells were exposed to LPS, intracellular inflammatory factors including NF‐κB p65 (*p* < 0.01), TNF‐α (*p* < 0.01) and IL‐6 (*p* < 0.01) overexpressed (Table 2). Besides, LPS also enhanced the expression of IDO1 (Figure 6D). E2 administration ameliorated the overexpression of the inflammatory factors and IDO1.

**TABLE 2:** Effects of E2 on NF‐κBp65, IL‐6 and TNF‐α expressions in BV2 cells.

Group	NF‐κBp65 (ng/mL)	IL‐6 (pg/mL)	TNF‐α (pg/mL)
Control	20.4 ± 3.7	2.4 ± 0.5	1.5 ± 0.4
ERα siRNA	19.9 ± 4.1	2.3 ± 0.6	1.4 ± 0.3
ERβ siRNA	20.2 ± 2.9	2.3 ± 0.9	1.6 ± 0.2
LPS	56.1 ± 5.4^a^	5.8 ± 1.0^a^	5.3 ± 0.5^a^
LPS + E2	30.4 ± 3.8^b^	3.6 ± 0.75^b^	2.8 ± 0.5^b^
E2 + ERα siRNA	31.0 ± 4.5	3.4 ± 0.4	2.4 ± 0.4
E2 + ERβ siRNA	42.7 ± 7.5^d^	4.9 ± 0.8^c^	4.2 ± 0.7^c^

*Note*: Values are expressed as mean ± S.D. *n* = 6. Data analysis was performed using Tukey's Test.

^a^ *p* < 0.01 versus the control group.

^b^ *p* < 0.01 versus LPS group.

^c^ *p* < 0.05 versus the LPS + E2 group.

^d^ *p* < 0.01 versus the LPS + E2 group.

Whereas ERβ siRNA pretreatment prohibited the function of E2 in BV2 cells (Table 2 and Figure 6D). In consideration of the result that the inflammatory factors were elevated by ERβ siRNA (Table 2) and the fact that IDO1 can be stimulated by inflammatory factors in microglia, the regulatory effect of ERβ on IDO1 may through modulating the inflammation. Interestingly, the expression of IDO1 was also significantly enhanced when the cells were treated with ERβ siRNA alone (*p* < 0.05, Figure 6E), indicating that ERβ could also directly regulate the expression of IDO1.

### 3.7 Effects of ERα/β siRNA on the LPS‐induced inflammatory response and H_2_O_2_ ‐inducedoxidative stress injury in HT22 cells

HT22 cells were used to investigate the effects of ERα/β siRNA on H_2_O_2_‐induced cellular oxidative stress as well as LPS‐induced inflammatory response.

**FIGURE 7:**
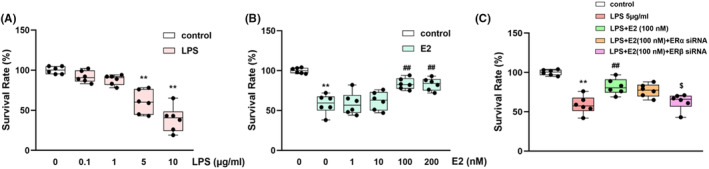
Survival rates of HT22 cells challenged with (0.1, 1, 5, 10 μg/mL) LPS administration (A). Effects of E2 (1, 10, 100, 200 mM) on the survival rate of TH‐22 cells challenged with 5 μg/mL LPS administration (B). ERβ siRNA treatment, not ERα siRNA, reversed the effects of E2 (100 mM) on LPS‐induced cell damage (C). Values are expressed as the mean ± S.D. with 6 mice in each group. **p < 0.01 compared with the control group; ^##^ *p* < 0.01 compared with the LPS group. ^$^ *p* < 0.05 compared with the E2‐treated LPS group.

The appropriate concentration of LPS used in HT22 cells was determined by testing a series of LPS concentrations in HT22 cells. The concentration of 5 μg/mL, which achieves a cell survival rate of 55.1 ± 7.2% (Figure 7A), was selected as the LSP concentration used for administration.

In this study, 100 nM and200nM showed similar improvement effects on the LPS‐induced decrease in cell viability (*p* < 0.01, Figure 7B), and the concentration of 100 nM was selected for administration in further experiments. ERβ siRNA pretreatment reversed the protective effect of E2 on cell survival (*p* < 0.05, Figure 7C). In addition, 100 nM E2 decreased the levels of LPS‐induced overexpression of inflammatory mediators (*p*s <0.01 for NF‐κB, TNF‐α and IL‐6, Table 3), while ERβ siRNA pretreatment, not ERα siRNA, reversed the anti‐inflammatory effect of E2.

**TABLE 3:** Effects of E2 on IL‐6 and TNF‐α expressions in HT‐22 cells.

Group	IL‐6 (pg/mL)	TNF‐α (pg/mL)
Control	22.4 ± 3.7	12.9 ± 1.6
LPS	45.2 ± 7.9^a^	26.9 ± 2.6^a^
LPS + E2	29.4 ± 5.1^b^	17.0 ± 2.4^b^
E2 + ERα siRNA	30.0 ± 4.7	16.8 ± 2.7
E2 + ERβ siRNA	39.2 ± 3.1^c^	22.4 ± 3.7^c^

*Note*: Values are expressed as mean ± S.D. *n* = 6. Data analysis was performed using Tukey's Test.

^a^ *p* < 0.01 versus the control group.

^b^ *p* < 0.01 versus LPS group.

^c^ *p* < 0.05 versus the LPS + E2 group.

**FIGURE 8:**
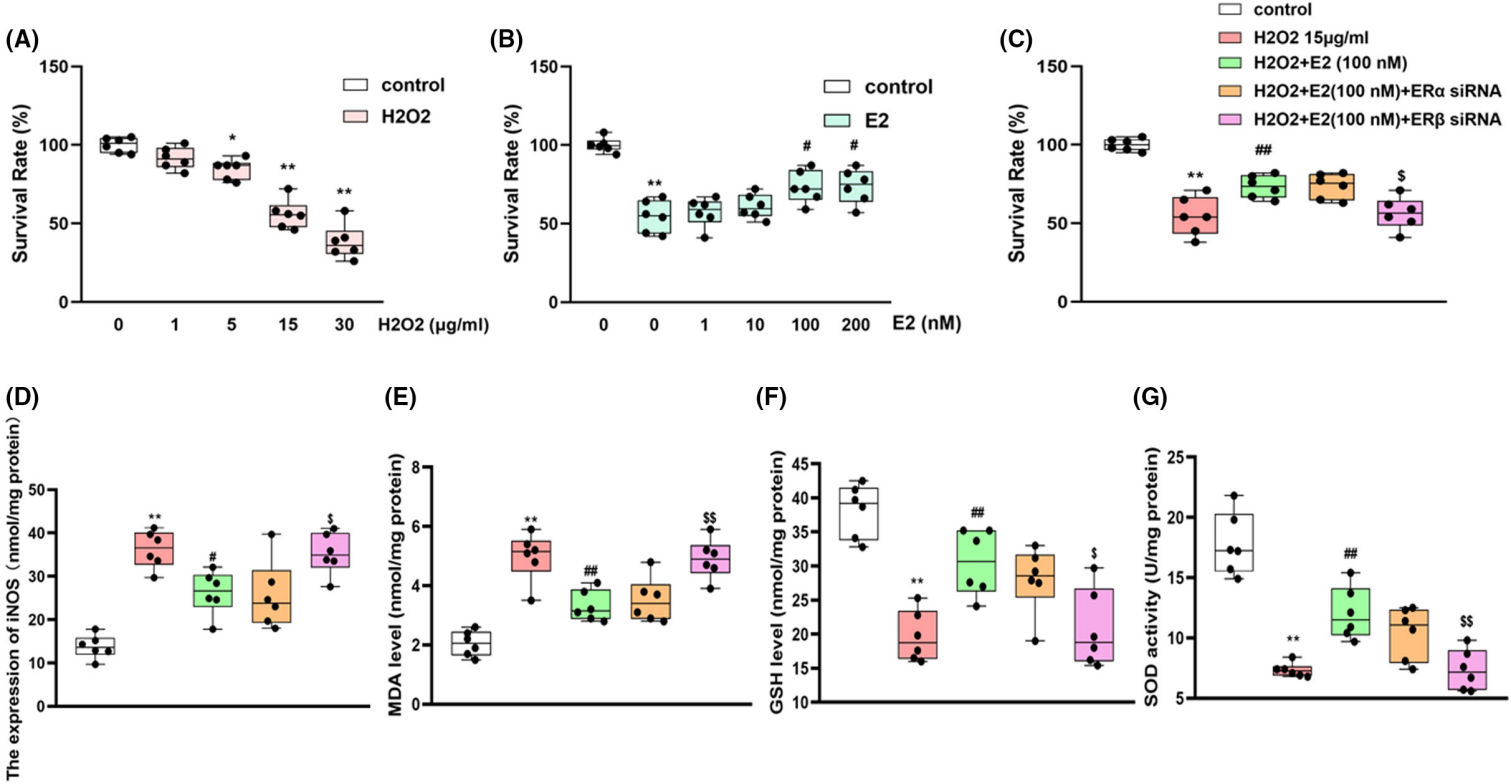
Survival rates of the HT22 cells challenged with H_2_O_2_ administration (1, 5, 15, 30 μg/mL) (A). Effects of E2 (1, 10, 100, 200 mM) on the survival rate of TH‐22 cells challenged with 15 μg/mL H_2_O_2_ administration (B). ERβ siRNA treatment reversed the effects of E2 (100mM) on H_2_O_2_‐induced cell damage (C). E2 treatment reversed the H_2_O_2_‐induced increase of iNOS (D) and MDA (E) levels and decrease of GSH (F) and SOD (G) levels. Values are expressed as the mean ± S.D. with 6 mice in each group. *p < 0.05 and **p < 0.01 compared with the control group; ^#^ *p* < 0.05 and ^##^ *p* < 0.01 compared with the H_2_O_2_ group. ^$^ *p* < 0.05 and ^$$^ *p* < 0.01 compared with the E2‐treated H_2_O_2_ group.

The appropriate concentration of H_2_O_2_ used in HT22 cells was determined by conducting a H_2_O_2_ tolerance experiment. The concentration of 15 μg/mL, which achieves a cell survival rate of 52.7 ± 5.1% (Figure 8A), was selected as the H_2_O_2_ concentration used for administration.

As for the concentration of E2, 100 nM/200 nM showed similar improvement effects on H_2_O_2_‐induced decrease in cell viability (*p* < 0.05, Figure 8B), and 100 nM was selected for administration in further experiments. ERβ siRNA pretreatment reversed E2's function on cell viability (*p* < 0.05, Figure 8C). Furthermore, E2 significantly improved the H_2_O_2_‐induced oxidative stress by decreasing the overexpression of iNOS (*p* < 0.05, Figure 8D) and MDA (*p* < 0.01, Figure 8E), and increasing the levels of GSH (*p* < 0.01, Figure 8F) and SOD (*p* < 0.01, Figure 8G). While ERβ siRNA pretreatment reversed the anti‐oxidation effects of E2 on HT22 cells.

### 3.8 Effects of E2 on LPS‐induced depressive‐like behaviour in male mice

**FIGURE 9:**
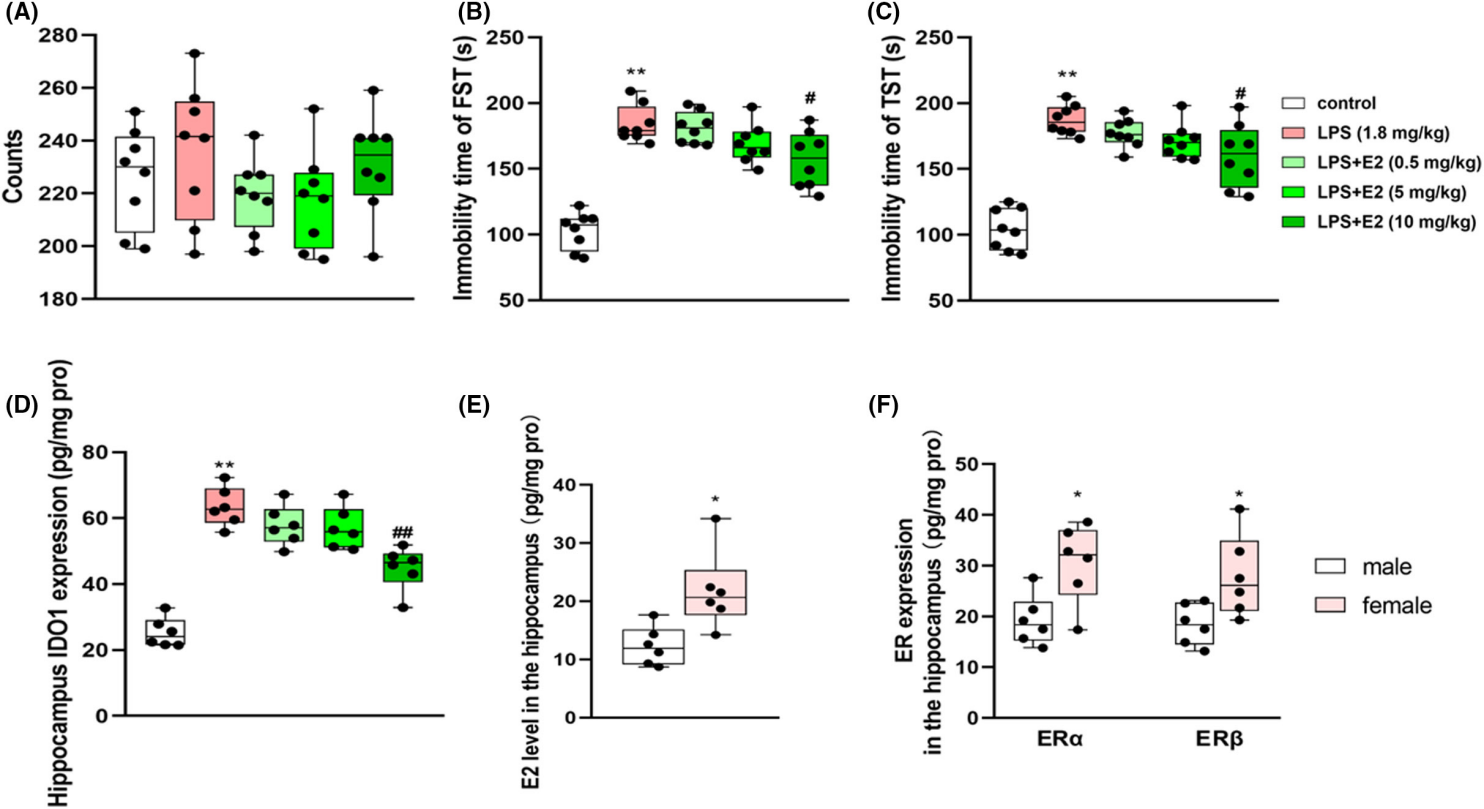
Effects of E2 on locomotor activity (A), forced swimming test (B) and tail suspension test (C) in LPS‐treated male mice. Effects of E2 on the IDO1 expressions in the hippocampus of male mice (D). Values are expressed as the mean ± S.D. with 8 mice in each group. **p < 0.01 compared with the control group; ^#^ *p* < 0.05 and ^##^ *p* < 0.01 compared with the LPS group. The expression of E2, ERα and ERβ in the hippocampus of male and female mice (E, F). *n* = 6 per group. *p < 0.05 compared with the male mice.

To investigate whether there is a difference in the function of E2 between females and males, we further evaluated the effect of E2 on male mice. As shown in Figure 9, LPS (1.8 mg/kg) resulted in significant depression‐like behaviours in male mice (*p* < 0.01 for FST and TST, Figure 9B,C). Besides, significantly increased levels of TNF‐α (*p* < 0.01, Table 4), IL‐6 (*p* < 0.01, Table 4), and IDO1 were found in hippocampal brain regions (*p* < 0.01, Figure 9D). E2 showed significant improvement effects on the depressive‐like behaviours (*p*s <0.05 for FST and TST), and the overexpression of TNF‐α, IL‐6 and IDO1 when the dose reached 10 mg/kg (20 times the dose in female mice). No significant difference was found in the spontaneous activity of animals among all the treatment groups (Figure 9A). In addition, our results revealed that the levels of E2, Erα and ERβ in the hippocampus were higher in female than that in male mice (*p*s <0.05 for E2 ERα and ERβ, Figure 9E–9F). Thus, we hypothesize that the differences in the efficacy of E2 on depression between male and female mice may be related to the different levels of E2 and ER in the brain between male and female mice, while the specific detailed mechanisms still need to be further demonstrated.

**TABLE 4:** Effects of E2 on IL‐6 and TNF‐α expressions in male mice.

Group	IL‐6 (pg/mg)	TNF‐α (pg/mg)
Control	19.7 ± 1.6	6.9 ± 0.9
LPS	41.4 ± 3.4^a^	15.2 ± 1.5^a^
LPS + 0.5 mg/kg E2	38.6 ± 2.3	14.8 ± 1.9
LPS + 5 mg/kg E2	36.6 ± 3.1	12.8 ± 2.1
LPS + 10 mg/kg E2	29.0 ± 5.6^b^	9.8 ± 1.6^b^

*Note*: Values are expressed as mean ± S.D. *n* = 6. Data analysis was performed using Tukey's Test.

^a^ *p* < 0.01 versus the control group.

^b^ *p* < 0.01 versus the LPS group.

To further explore whether the effect of E2 on male mice was realized through the agonism of ERβ, we co‐administrated ERβ inhibitor in E2‐treated male mice. The results showed that high‐dose ERβ inhibitor co‐administration reversed the antidepressant effect of E2 in male mice (Figure S1A–C) as well as E2's impact on IDO1 expression (Figure S1D), suggesting that the effect of E2 on LPS‐induced depressive‐like behaviours in male mice may be achieved through the E2/ERβ pathway, while further study is needed to confirmed whether E2's modulation is the same as that in female mice.

## 4 DISCUSSION

Older women, especially those in the menopause stage, have a high risk of depression.^25^ The onset of depression in menopause may be due to multiple reasons and oestrogen withdrawal is a critical one.^25^ Studies have shown that E2 is effective in treating major or minor depression in perimenopausal women.^26^, ^27^ However, oestrogen treatment increased risks of certain cancers, and progesterone may compromise the psychological benefits of oestrogen treatment.^5^, ^6^ Therefore, it is still important to study the mechanisms by which E2 ameliorates oestrogen deficiency‐induced depression for the development of effective treatment with mild adverse effects.

In this study, an OVX animal model was established to simulate the physiological characteristics of oestrogen withdrawal in human based on a previous research,^28^ and the depressive symptoms of mice were evaluated by FST and TST, both of which use the immobility time as the main indicator to evaluate the behaviour of animals in extreme environments.^28^ The results showed that the immobility time significantly increased and serum E2 levels decreased in OVX mice, indicating that the animal model was successfully established.

NF‐κB, a nuclear transcription factor, regulates various proinflammatory cytokines, such as IL‐1β, IL‐6 and TNF‐α.^29^, ^30^ A malignant increase of these genes in the brain can cause behavioural abnormalities, mood and sleep fluctuation and learning and memory deterioration.^31^ In our study, the hippocampal inflammatory mediators including NF‐κB, IL‐6 and TNF‐α significantly increased in OVX mice, which was in consistent with a previous study that OVX increase NF‐κB expression and further stimulate the release of inflammatory mediators.^6^ Previous studies found that E2 can improve inflammation‐induced depression‐like behaviour by suppressing neuroinflammation.^13^ This finding was substantiated by our results that the overexpressed anti‐inflammatory mediators and the immobility time in behaviour tests decreased after E2 administration and E2 inhibitor reversed this phenomenon. In BV12 cells, the increased levels of NF‐κB, IL‐6 and TNF‐α were decreased after E2 treatment while ERβ siRNA pretreatment reversed E2's action, further demonstrating that the inhibition of NF‐κB‐mediated inflammatory pathway is a critical mechanism of E2's antidepressant function.

**FIGURE 10:**
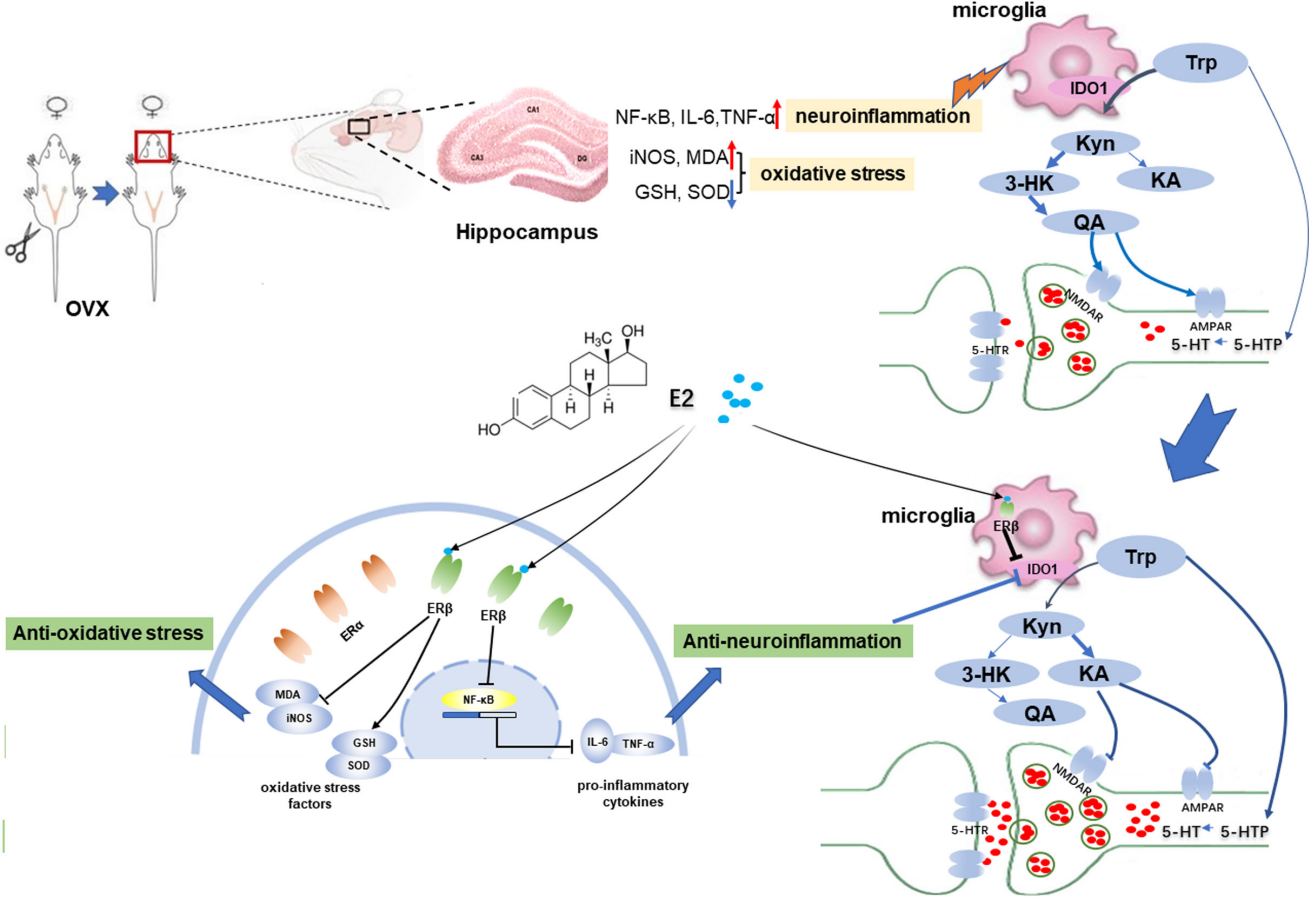
Molecular mechanisms involved in the protective effects of E2 against OVX‐induced depression. The activation of NF‐κB mediated neuroinflammation and the subsequent activation of IDO1 are involved in the pathomechanism of OVX‐induced depression. IDO1 in the hippocampus can be activated by either proinflammatory cytokines (e.g. TNF‐α, IL‐6, IL‐1β) or directly by ERβ activation in microglia. IDO1 activation results in decreased 5‐HT/TRP, which induces the decrease in the ratio of 5‐HIAA/5‐HT, triggering individual symptoms of depression and the increase of KYN/TRP ratio, which induces the imbalance of KYN metabolism. Normally, KYN is metabolized to kynurenic acid (KA), an NMDA antagonist. When IDO1 is activated, KYN is more likely metabolized to quinolinic acid (QA), a potent N‐methyl‐D‐aspartate (NMDA) receptor agonist and may be a key contributor to the increased neurotoxicity. In addition, improvement of oxidative stress (e.g. decrease of MDA and iNOS, increase of SOD and GSH) is also involved in the anti‐neurotoxicity effect of E2 against OVX‐induced depressive‐like behaviours. E2 can exert antidepressant through the activation of ERβ, by which modulating neuroinflammation, IDO1‐mediated tryptophan (TRP) metabolism and oxidative stress.

IDO1 is a subtype of IDO widely distributed in neurons of the hippocampal region.^32^ In the normal body, IDO1 is inactive and TRP plays a neuroprotective role and regulates mood, mainly through the 5‐HT metabolic pathway.^12^, ^18^ When the body is in a state of inflammation, inflammatory factors activate IDO1, and then a large number of neurotoxic substances, such as 3‐HK and QA, are produced from TRP metabolism in the KYN pathway.^12^, ^18^ Relevant studies have shown that the activation of IDO1 induced by inflammation in the brain is related to the neuroinflammation‐induced activation of microglia.^33^, ^34^ Under different environmental stimuli, microglia can be classically activated (M1 phenotype) or alternatively activated (M2 phenotype).^35^ Microglia with the M1 phenotype have proinflammatory effects, which are mainly induced by inflammatory inducers such as LPS and secrete a large number of proinflammatory cytokines, including TNF‐α, IL‐1β and IL‐6.^36^ Activated microglia under normal circumstances help to clear invading pathogens and necrotic cells. Once microglia are overactivated, M1 phenotype microglia release high levels of the abovementioned inflammatory factors and neurotoxins, resulting in an uncontrolled inflammatory response and further activation of IDO1, resulting in changes in the metabolic pathway of TRP, from the 5‐HT metabolic pathway to the KYN metabolic pathway.^37^ The M2 phenotype is induced by IL‐4/IL‐3 to produce high levels of anti‐inflammatory factors such as IL‐10, transforming growth factor beta (TGF‐β) and glucocorticoids (GCs).^35^ M2 phenotype microglia are considered protective cells that secrete anti‐inflammatory factors and upregulate neuroprotective factors. In our correlation analysis, IDO1 was positively correlated with the immobility time in the behaviour tests and negatively correlated with the E2 level in serum, indicating that IDO1 is likely to be involved in the pathomechanism of oestrogen withdrawal‐induced depression. This was confirmed by our results that IDO1 inhibitor 1‐MT administration reversed the immobility time in FST and TST. In combination with the result that the hippocampal inflammatory mediators significantly increased in OVX mice, it can be speculated that the IDO1 level was triggered by increased hippocampal inflammation after oestrogen withdrawal. The changes of IDO1 overexpression and the downstream neurotoxic substances of the KYN pathway were in consistent with that of the inflammatory mediators after E2 treatment, and E2's inhibitor reversed the changes, indicating that E2 may exert the antidepressant function in OVX mice via inhibiting neuroinflammation, thereby inhibiting the IDO1‐mediated TRP/KYN pathway. This speculation was also supported by the results of our experiments in microglial cells BV2. Previous studies have found that E2 can improve the hyperactivation of BV2 microglia induced by hypoxia and ischemia, transform M1 to M2 microglia and exert anti‐inflammatory and neuroprotective effects.^35^ Our cell experiments have shown that E2‐mediated activation of ERβ inhibited LPS‐induced overexpression of inflammatory factors, and then inhibited the activation of IDO1. Interestingly, in the absence of LPS stimulation, silencing ERβ directly led to a reduction in IDO1 levels, suggesting that E2‐mediated ERβ activation can not only indirectly reduce IDO1 expression by inhibiting the inflammatory response but may also have a direct regulation effect on IDO1. However, the regulatory effects of ERβ on microglia and IDO1 need to be further verified in vivo (Figure 10).

In addition to the neuroinflammation and IDO1 activation, oxidative stress is also a mechanism involved in the pathophysiology of depression.^36^, ^37^ Prooxidants and antioxidants are in balance under normal biological systems; however, an increase in oxidants and a decrease in antioxidants causes imbalance, eventually leading to oxidative stress.^37^ Relevant studies have shown that the antioxidant defence system (e.g. SOD and GSH) were suppressed and the oxidants (e.g. ROS and MDA) increased in OVX mice.^16^, ^38^ In this study, OVX caused significant oxidative stress damage in the hippocampal region and abnormal SOD, GSH, MDA and iNOS expression were improved by E2. Moreover, antagonizing ERβ receptors can reverse the antioxidative stress effect of E2 in both in vivo and in vitro experiments. These results suggested that the antidepressant effect of E2 is related to the regulation of oxidative stress factors by the ERβ pathway in the hippocampus.

Interestingly, our study found that inhibition of ERα has no significant effect on neuroinflammatory mediators, IDO1 activity and oxidative stress factors, suggesting that ERα may not be involved in E2's effect on OVX‐induced depression. In the central nervous system, there are various ER subtypes, of which ERα and ERβ are highly expressed. The two ERs have different distribution patterns and different physiological processes in the brain, leading to different and possibly contradictory roles of the two receptors.^39^ In the hippocampus, the regulatory effect of ERβ on neuroinflammation and oxidative stress is more dominant, when compared to ERα. The difference in the regulatory effects of ERα/ERβ on inflammatory factors and oxidants has been further confirmed in cell experiments. ERβ siRNA pretreatment reversed the anti‐inflammatory effect and antioxidant effect of E2 in the LPS‐treated nerve cells, while ERα siRNA had no significant effect.

It is worth mentioning that 0.5 mg/kg E2 had no effect on depressive‐like behaviours, but 10 mg/kg (20 times the dose of female mice) E2 caused significant improvement in depression of the male mice, suggesting that the endogenous E2 levels between female and male mice are different, resulting in the different regulatory intensities on inflammatory factors and IDO1 activity. A relevant study also revealed that oestrogen plays a different role in male and female.^40^

## 5 CONCLUSION

The results of this study demonstrated that IDO1 in the hippocampus is involved in the depressive‐like behaviours caused by oestrogen withdrawal. Oestrogen has antidepressant function, the mechanisms of which include inhibiting the NF‐κB‐mediated inflammatory pathway and suppressing the release of oxidants and enhancing the levels of antioxidants. Importantly, we innovatively found that oestrogen may inhibit neurotoxic substances by affecting the IDO1‐mediated KYN pathway, either directly by regulating ERβ in BV2 cells or indirectly by regulating the inflammatory factors. The antidepressant function of oestrogen is achieved via the activation of ERβ, but not the ERα. To the best of our knowledge, this is the first study exploring the role of oestrogen in the IDO1‐mediated KYN metabolic pathway in the central nervous system. We believe the findings in this study may provide valuable information for the development of antidepressant therapies.

## AUTHOR CONTRIBUTIONS

**Xi Jiang:** Writing – original draft (equal). **Xuefeng Yu:** Writing – original draft (equal). **Shuran Hu:** Formal analysis (equal); investigation (equal). **Huidan Dai:** Formal analysis (equal); investigation (equal). **Hanqin Zhang:** Formal analysis (equal); investigation (equal). **Yuyang Hang:** Investigation (equal). **Xupei Xie:** Formal analysis (equal); investigation (equal). **Fan Wu:** Conceptualization (lead). **Yubo Yang:** Data curation (equal).

## FUNDING INFORMATION

This research was financially supported by the Science and Technology Project of Ningbo (202002 N3151) for Xuefeng Yu; Technology Project of Zhejiang Medical and Health Department (2024KY494) for Xupei Xie and (2023RC197) (2024ZR070) for Fan Wu; the Science and Technology Project of Zhejiang Medical and Health Department (2020KY289), the Science and Technology Project of Ningbo Yinzhou District (2022AS036), Ningbo Natural Science Foundation (202503 N4268) for Xi Jiang; Key Discipline Projects in Yinzhou District of Ningbo for Yubo Yang and Xi Jiang.

## CONFLICT OF INTEREST STATEMENT

The authors state that they have no conflicts of interest related to this publication.

## Supporting information


Figure S1.

## Data Availability

The data of this study can be obtained from the corresponding author upon reasonable request.
