# Supplementary figures and images for: Effects of E2 on the IDO1‐mediated metabolic KYN pathway in OVX female mice
Source: J Cell Mol Med. 2024 Oct 28;28(20):e70179. doi: 10.1111/jcmm.70179 (PMC11518696; doi:10.1111/jcmm.70179)

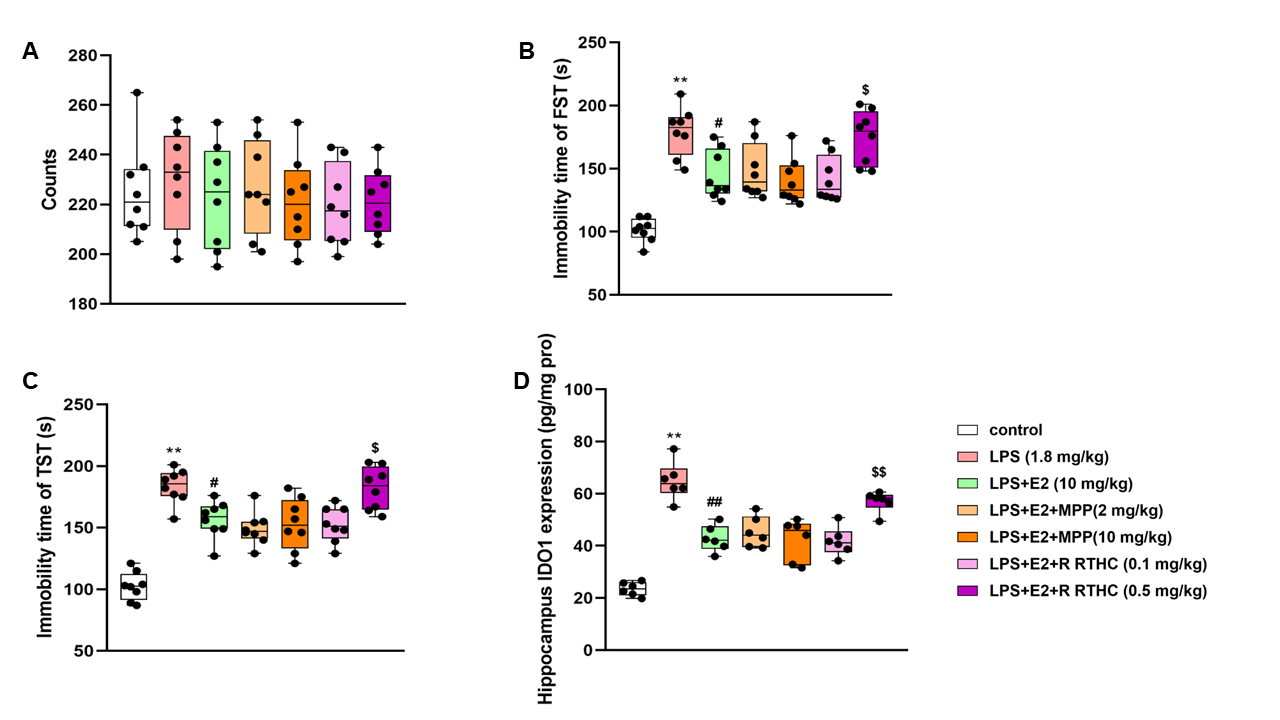

Supplement: Supplementary file 1 — Figure S1. [file JCMM-28-e70179-s001.zip › jcmm70179-sup-0001-FigureS1.TIF]
